# Household income and children’s mental health outcomes: the mediating role of maternal wellbeing and parent–child relationship quality

**DOI:** 10.1007/s00787-025-02765-y

**Published:** 2025-06-07

**Authors:** Naomi Wilson, Helen Minnis, Shari McDaid, Anna Pearce

**Affiliations:** 1 University of Glasgow, Clarice Pears Building, 90 Byres Road, Glasgow, G12 8 TB Scotland; 2 Mental Health Foundation, Glasgow, SC 039714 Scotland

**Keywords:** Inequalities, Child and adolescent psychiatry, Attachment, Public health

## Abstract

Uncertainty remains about the mechanism(s) through which household income influences mental health across the life course. The family stress model suggests the role of parental mental health and child-parent relationship quality may be significant, however few studies have explored this longitudinally. The present study aimed to decompose the pathways from low household income at age 3 to children's mental wellbeing at age 6, via maternal wellbeing (age 4) and parent–child relationship quality (age 5). We included four sweeps of the Growing Up in Scotland Study (*n* = 3639). Weights and multiple imputation were used. Low income was defined as the two bottom quintiles of equivalised household income. Potential mental health difficulties were determined using the Strengths and Difficulties Questionnaire. Mediators were assessed using the Depression, Anxiety and Stress Scale and the Pianta Child-Parent Relationship Scale. A causal mediation analysis was conducted. Confounding factors adjusted for included ethnicity, family type, maternal age, education and employment. Children from low-income households at age 3 were more than twice as likely to have a potential mental health difficulty at age 6 compared to all other children (adjusted odds ratio (OR) 2.16 (95% CI 1.50 to 3.80). Reduced maternal wellbeing mediated 11.21% of the total effect. Adding perceived child-parent relationship quality to this model increased the proportion mediated to 30.89%. Increasing the incomes of young families will improve child mental health. Given the long-term risks associated with childhood mental health difficulties this should be a public health priority.

**Supplementary Information:**

The online version contains supplementary material available at 10.1007/s00787-025-02765-y.

## Introduction

The association between growing up in a low-income household and experiencing mental health difficulties in childhood is well established [1]. Children growing up in homes with fewer financial resources have repeatedly been found to report lower subjective well-being [2, 3] and to be at an increased risk of anxiety, depression, and a range of other mental health disorders in later life [4]. There is also evidence to show that this association is causal in nature, with studies repeatedly demonstrating that household income has a positive causal effect on children’s outcomes, including their cognitive and social-behavioural development and health [5]. Despite this, there remains little conclusive evidence on the mediators or mechanism(s) through which household income influences mental health across the life course [5]. Such uncertainty leaves room for considerable difference of opinion about policy solutions. For example, current income supplementation policies, such as Universal Credit (presently the main form of income support for those who are out of work or living on a low income in the United Kingdom), have not been shown to ubiquitously improve mental health outcomes [6, 7], and conversely have been identified in some studies to have a detrimental impact on the mental health of recipients [8, 9]. Such insights highlight the need for further research on the causal mechanisms linking income and mental health [10]. Moreover, while child poverty reduction policies are being implemented, these are unlikely to wholly eliminate child poverty nor create equality across incomes [11]. Understanding the additional amenable pathways through which income influences mental health across development could therefore highlight where additional interventions should be focused.

**Fig. 1:**
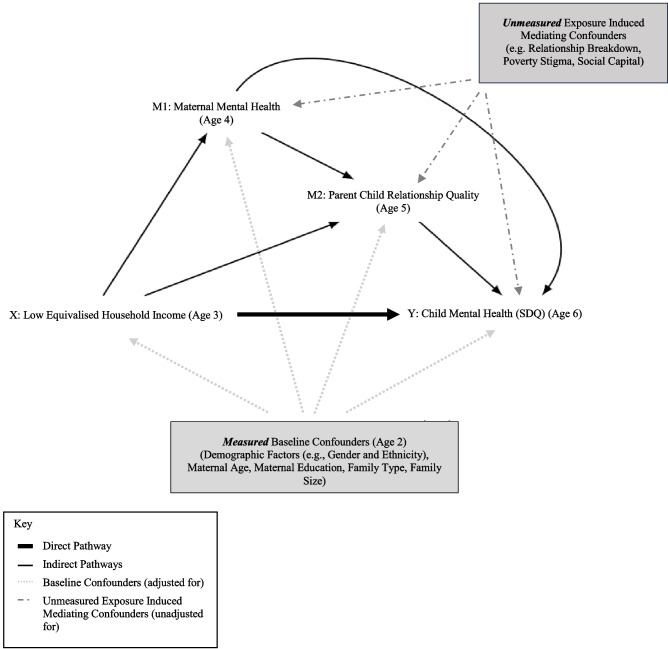
Simplified directed acyclic graph of hypothesized association between equivalised household income (Age 3) and child mental health (Age 6)

The mediating pathways between household income and children’s mental health outcomes are likely to be complex [5]. A range of behavioural and psychosocial factors have been proposed to interact over the course of child development to influence risk of future mental health difficulties through multiple simultaneous pathways [12]. However, the ‘family stress model’ is an increasingly influential theoretical framework which attempts to account for one of these pathways [13].Originally developed by Conger and colleagues in 1992, the model suggests that economic stress—such as low income, job loss, or financial strain—leads to reduced parental wellbeing, which in turn affects parenting behaviours and, ultimately, child outcomes [14, 15]. Specifically, according to this model, financial difficulties contribute to emotional distress in parents (including depression and anxiety), which reduces the emotional resources they have available for supportive and nurturing parenting behaviours [14, 15]. This in turn negatively impacts household relationships, including parent–child relationships, and can result in less responsive and more harsh or inconsistent parenting, which consequently influences children's emotional and behavioural development, including their later mental health outcomes [13]. A visual representation of this model, and of the proxy measures used for the components assessed in the current study is provided in Supplementary Material (Fig. 1).

The role of the first mediator in this model (i.e. parental wellbeing) is partially supported by previous studies from within the UK which have identified that the mediating role of maternal mental health problems are of particular importance to children’s mental health outcomes, in the context of economic disadvantage [16, 17]. The role of the second mediator (i.e. parent child relationship quality) is also supported by growing evidence from out-with the UK to suggest that at least some of the association between economic hardship and children’s mental health outcomes may be mediated via the impact of poverty on the quality of parent–child relationships [18, 19]. Specifically, several North American studies have found social welfare policies that increase household income without disrupting the amount of time parents are able to spend with their children can lead to significantly improved childhood mental health outcomes, through improving parent–child relationships [20, 21]. However, since cultural and contextual factors are likely to be significant, to accurately inform policymakers, it is crucial to generate robust longitudinal evidence on this complete pathway amongst samples of children in other high-income countries such as the UK.

Household incomes are affected by the presence, number, and age of children, with families needing a higher income to maintain a similar standard of living as their children progress from infancy into early childhood and beyond [22]. The impact of exposure to low income is also known to vary across development [5] and determining its specific effects at variety of ages is therefore necessary for tailoring policy responses. While an increase in poverty across the United Kingdom has been observed across all sociodemographic groups in recent years, the steepest rise has been observed in families where at least one parent is in work, and among those with children under the age of 5 [23]. Exploring the effect of exposure to low income in early childhood (typically defined as between 3 and 8 years of age [24]) specifically is therefore crucial for informing targeted policy solutions.

The aim of the current study was to decompose the pathways between low household income in early childhood (age 3) and children’s mental health outcomes in middle childhood (age 6) to determine the extent to which this association is: (a) *indirectly* mediated via maternal wellbeing (age 4) and parent–child relationship quality (age 5) (acting in isolation and sequentially); and (b) *direct* (i.e., acts through mechanisms that bypass these putative mediators), while controlling for a range of confounding factors (Fig. 1).

## Methods

### Participants

Data were from the first birth cohort of the Growing Up in Scotland (GUS) study, a nationally representative cohort of 5217 children, and their families, born between June 2004 and May 2005. Details of the sampling framework are provided elsewhere [25]. Our sample included data collected at sweeps 2–6, when the children were 2, 3, 4, 5 and 6 years of age, and where the mother reported information relating to both mediators (*n* = 3639). This was to ensure it was mother’s mental wellbeing (at sweep 4) and mothers’ perceptions of their child-parent relationship quality (at sweep 5) which was measured for all participants (as opposed to any other household members reports on either of these scales).

Response rates at each sweep are provided in Supplementary Table 1, however these were 80% in Sweep 1, with loss to follow-up in subsequent included sweeps ranging from 14 to 30%.

### Ethics

GUS baseline data collection was subject to medical ethical review by the Scotland ‘A’ MREC committee (application reference: 04/M RE 1 0/59) and via substantial amendment submitted to the same committee for subsequent sweeps. All participants provided written informed consent. Further consent and ethical approval were not required for the secondary analyses presented in this paper.

### Patient and public involvement and engagement

This work was co-produced with the Mental Health Foundation (Scotland SC 039714) a third sector organisation who works closely with experts by experience and focuses on expanding the evidence base for mental health prevention strategies. Specifically, MHF contributed to the study design, inclusion of covariates, and interpretation of results.

### Measures

The counterfactual analytical method used to decompose the mediating pathways of interest (see 2.7 Statistical Analysis) favours the use of binary exposure, mediator and intermediate confounding variables, because the availability of just one counterfactual state aids interpretability of results [26]. Therefore, results for all measures were dichotomised.

#### Exposure: equivalised household income (Age 3, Sweep 3)

Self-reported household income was collected via interview at each GUS sweep. This was then equivalised using the standardised OECD (Organisation for Economic Co-operation and Development) modified equivalence scale, which adjusts household income to take account of the differences in a household's size and composition [27]. Children in the first and second quintile groups, representing the 40% of GUS with the lowest equivalised income (or an equivalised household income < 60% of the median) at 3 years of age (sweep 3 of GUS), were considered ‘exposed’ to ‘low’ household income. Those in all other income quintiles at age 3 were considered to be in a middle or high income group and were therefore collapsed into an ‘unexposed’ group.

#### Outcome: childhood mental health difficulties (Age 6, Sweep 6)

The Strengths and Difficulties Questionnaire (SDQ) completed by the child’s primary caregiver when children were 6 years of age (sweep 6 of GUS), was used as a measure of children’s emotional and behavioural wellbeing. This 25-item behavioural screening questionnaire is designed for use with 3–16-year-olds. It has previously been shown to be reasonably reliable in identifying children with a diagnosed mental health difficulty in the community [28] (with Cronbach’s alpha values ranging from 0.63 to 0.88 [29]) and to differentiate well between clinical and non-clinical samples in a number of studies [30]. It contains 5 subscales (emotional symptoms, conduct problems, hyperactivity/inattention, peer relationship problems, and prosocial behaviour) and provides a “Total Difficulties Score” comprising the 20 “problem” items (excluding the prosocial behaviour subscale), with higher scores corresponding to a greater risk of mental health disorder [31]. Validated cut-offs for normal (< 14), borderline (14 to 16) abnormal (≥ 17) are provided [32]. Therefore, scores of 17 or above were utilised to identify incidents of potential “childhood mental health difficulties”.

#### Mediator 1: reduced maternal mental wellbeing (Age 4, Sweep 4)

**Fig. 2:**
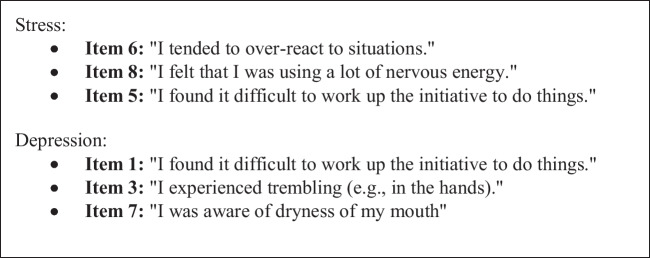
Selected DASS items used in GUS

Within GUS maternal mental health was assessed using 6 items from the Depression, Anxiety and Stress Scale (DASS) (3 items for depression and 3 items for stress) (Fig. 2). The DASS is a validated, widely used scale, which has been shown to have good reliability in community-based samples [33]. Responses to each item are summed, with higher scores being indicative of increasing symptoms of anxiety and depression. No validated ‘cut-off’ scores for the 6 questions selected from DASS are available [34]. Therefore, in order to make a dichotomised variable, those with scores in the highest quintile were considered to have reduced maternal wellbeing, while all other scores were considered to be within normal range.

#### Mediator 2: child-parent relationship quality (Age 5, Sweep 5)

The Pianta Child-Parent Relationship Scale-Short Form (CPRS-SF) was used as a measure of the quality of the relationship between children and their primary caregiver. This was completed by the child’s primary caregiver when children were 5 years of age (sweep 5 of GUS). The CPRS-SF is a widely used self-report instrument that assesses parents’ perceptions of their relationship with their child [35] and has been shown to have acceptable internal consistency (Cronbach’s alpha 0.69–0.84) [36]. The short form consists of 15 items, 8 of which are summed into a conflict subscale and 7 of which are summed into a closeness subscale [37]. No validated cut-offs for these subscales are provided [36, 37]. Therefore, scores which were either in the lowest quintile for closeness, or the highest quintile for conflict were deemed indicative of ‘poor’ child-parent relationship quality.

### Covariates

Baseline confounders were chosen on the basis of common causes of exposure and: the mediators and/or outcome [38]. These were drawn from data collected at age 2 (sweep 2 of GUS) and included: ethnicity (white UK vs ethnic minority); family type (one or two parent household); household size (number of children living in the household); mother’s age at first live birth (< 20, 20–29, 30–39, > 40); maternal education (no qualifications, compulsory educational qualifications [i.e. Scottish Standard Grades or equivalent], Scottish Higher Qualifications or equivalent, or any form of further education); and maternal employment (whether the mother was in any kind of paid employment or not).

### Missing data

Missing data for exposures, mediators, outcomes, and baseline confounders ranged from less than 0% to 11%. Of the individuals eligible for inclusion (3639), 3011 had no missing data for any of the variables included in our analysis. Little’s test of missing completely at random was first conducted in SPSS statistical software (Version 29.0.2.0) [39] to assess whether missingness was at random or was associated with variation of analysed variables. This suggested that data were not missing at random. Multiple imputation through chained equations (MICE) using the R package MICE [40], was therefore used to minimize bias due to missing data (20 imputations using 50 iterations). The imputation models included all variables in the target analysis. Distributions of original and imputed data were similar when compared with t-tests and various density plot functions in the R package ‘VIM’ [41], suggesting that the multiple imputations produced plausible data for missing values.

### Statistical analysis

We undertook a causal mediation analysis under the counterfactual framework to partition the Total Effect (TE) of low equivalised household income at age 3 on mental health difficulties at age 6, acting through the proposed mediators (natural indirect effect (NIE)), both in isolation and sequentially, and through mechanisms that bypass these putative mediators (natural direct effect (NDE)) (Fig. 1). Our understanding of the temporal sequence of mediators and the timing of measurement led us to choose this sequential approach. Longitudinal survey weights provided within GUS were applied to account for the sample design and attrition. We estimated odds ratios (OR) and 95% confidence intervals (CI), using non-parametric bootstrapping for 1000 iterations, for the NDE, NIE and TE sequentially, using the *medflex* package in R (V 4.2.1) [42]. This parameterises the path-specific effects of interest in the presence of multiple mediators, taking into account potential interactions between the mediators of interest [42]. Finally, we estimated the proportion mediated (PM) in each model using the formula [43]:

$$\frac{{OR}^{NDE}({OR}^{NIE}-1)}{({OR}^{NDE} \times {OR}^{NIE}-1)}$$

This method does not allow for the adjustment of exposure-induced mediator outcome confounders. We consider the limitations of not accounting for this confounding in the discussion.

## Results

### Baseline sociodemographic characteristics

**Fig. 3:**
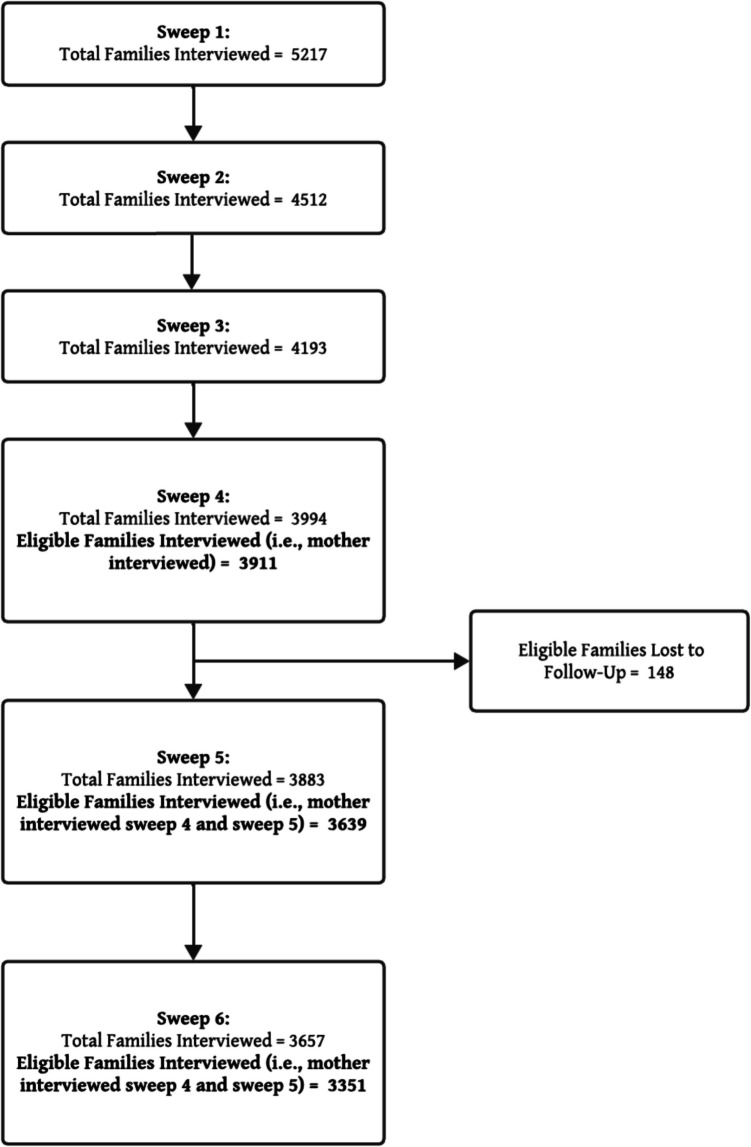
Participant flow diagram

A participant flow diagram and baseline sociodemographic characteristics are provided in Fig. 3 and Table 1 respectively. The mean age of children at baseline was 2.88 years (SD 0.37) and the majority resided in a two-parent household. Overall, 4.5% of mothers reported that at age 6 their child had sufficient difficulties that they were within the mental health difficulties range on the SDQ (scored ≥ 17). The majority of mothers were between 30 and 39 years of age, were in employment and had a university degree or vocational qualification. The imputed sample was generally similar to the non-imputed survey sample.

**Table 1:** Sociodemographic characteristics of sample (*n* = 3639) before and after imputation

Characteristics	Survey sample	Imputed sample
	*n* (%)	*n* (%)
Child Characteristics		
Sex		
Male	1834 (51.0)	1855 (51.0)
Female	1756 (49.0)	1784 (49.0)
Missing Cases	49	-
SDQ Score		
Normal	3020 (90.1)	3290 (90.4)
Borderline	169 (5.1)	177 (4.9)
Abnormal	162 (4.8)	172 (4.7)
Missing Cases	288	-
Maternal Characteristics		
Education		
Degree or Vocational Qualification	2503 (70.0)	2549 (70.0)
Scottish Higher Qualification	305 (8.5)	306 (8.5)
Scottish Standard Grade	540 (15.1)	550 (15.2)
No Qualifications	229 (6.4)	234 (6.3)
Missing Cases	62	-
Age (years)		
< 20	159 (4.4)	161 (4.4)
20—29	1300 (36.4)	1315 (36.1)
30—39	1986 (55.6)	2028 (55.7)
> 40	129 (3.6)	135 (3.7)
Missing Cases	65	-
Employment		
Employed	2360 (65.8)	2389 (65.6)
Unemployed	1229 (34.2)	1250 (34.4)
Missing Cases	50	-
Family Characteristics		
Number of Children in Household		
1	1510 (42.1)	1526 (41.9)
2–3	1943 (54.1)	1972 (54.2)
> 4	137 (3.8)	141 (3.9)
Missing Cases	49	-
Family Type		
One Parent Family	498 (14.0)	512 (14.1)
Two Parent Family	3059 (86.0)	3127 (85.9)
Missing Cases	82	-
Ethnicity		
White Caucasian	3491 (97.3)	3540 (97.3)
Other Ethnicity	97 (2.7)	99 (2.7)
Missing Cases	51	-
Equivalised Household Income		
Low	1278 (38.2)	1416 (38.9)
Medium/High	2071 (61.8)	2223 (61.1)
Missing Cases	290	-

### Childhood mental health difficulties (outcome), maternal wellbeing and child-parent relationship quality (mediators) according to equivalised household income

Table 2 shows that the prevalence of potential childhood mental health difficulties at age 6 was more than three times higher among children in the lowest two quintiles for equivalised household income at age 3 compared with all other children. Children from less economically advantaged backgrounds were also more likely to have a mother who described reduced wellbeing and who self-reported lower levels of perceived closeness or greater levels of perceived conflict in their relationship with their child, although differences were greater for maternal wellbeing. Differences in all baseline confounding factors were also observed.

**Table 2:** Children with normal, borderline and abnormal SDQ scores (outcomes), maternal mental health and child parent relationship quality (mediators), and confounding variables, according to low and high Equivalised Household Income Quintile: % (*N*), Odds Ratios (OR) (95% Confidence Intervals (CIs))

Exposure (X): Income	Low	Medium/High	Low vs. Medium/High	
	*N* (%)	*N* (%)	OR	(95% CI)
Outcome (Y)				
Abnormal SDQ	118 (8.3)	60 (2.7)	3.27	(2.38 to 4.50)
Mediators (M1 and M2)				
Reduced Maternal Wellbeing^1^	433 (30.6)	409 (18.4)	1.95	(1.67 to 2.28)
Child Parent Relationship Quality^2^	575 (40.6)	700 (31.5)	1.49	(1.29 to 1.71)
Baseline Confounding (C)				
Low Maternal Education^3^	523 (36.9)	261 (11.7)	4.40	(3.72 to 5.21)
Low Maternal Age^4^	133 (9.4)	28 (1.3)	8.13	(5.38 to 12.29)
Maternal Unemployment	749 (52.9)	501 (22.5)	3.86	(3.34 to 4.46)
> 4 children in household	98 (6.9)	43 (1.9)	3.78	(2.62 to 5.44)
One parent family	425 (30.0)	87 (3.9)	10.53	(8.26 to 13.42)
Ethnic minority	64 (4.5)	35 (1.6)	2.96	(1.95 to 4.49)

1. Highest quintile on DASS

2. Lowest quintile for closeness or highest quintile for conflict on CPRS-SF

3. Mother with no or only statutory qualifications (i.e. Scottish Standard Grades or Equivalent)

4. Mother < 20 years old at birth of study child

### Mediation analysis

The Total Effect (TE) of exposure to low equivalised household income at age 3 and mental health difficulties at age 6 (an Odds Ratio [OR] comparing children from the lowest two equivalised household income quintiles with all other children, while controlling for all identified confounding factors) was 2.16 (95% CI 1.50 to 3.80) (Table 3).

**Table 3:** Pathways between low equivalised household income at age 3 on mental health difficulties at age 6

Exposure	Effect and mediator(s)	OR	95% CI	PM
Low Household Income	Total Effect	2.16	1.50 to 3.80	-
	Natural Direct Effect	2.02	1.39 to 3.53	-
	Natural Indirect Effect via* MMH*	1.06	1.02 to 1.12	11.21%
	Natural Indirect Effect via* CPRQ*	1.11	1.05 to 1.19	18.26%
	Natural Joint Indirect Effect via* MMH and CPRQ*	1.19	1.12 to 1.33	30.89%

*OR* Odds Ratios; *CI* Confidence Intervals; *PM* Proportion Mediated; *MMH* Maternal Mental Health; *CPRQ* Child Parent Relationship Quality

The natural direct effect (NDE), that is the effect of equivalised household income on child mental health outcomes which was not mediated via either maternal mental health or child-parent relationship quality was 2.02 (95% CI 1.39 to 3.53). The indirect effect mediated via maternal mental health problems measured when the child was age 4 was 1.06 (95% CI 1.02 to 1.12) and the proportion mediated via this pathway was 11.21%. The indirect effect mediated via perceived child parent relationship quality measured when the child was aged 5 was 1.11 (95% CI 1.05 to 1.19) and the proportion mediated via this pathway was 18.26%. A combined pathway of maternal mental health and child parent relationship quality acting sequentially was found to mediate 30.89% of the association between low equivalised household income at age 3 and mental health difficulties at age 6 (Table 3).

## Discussion

The aim of the present study is to decompose the pathways from low household income in early childhood to children's mental wellbeing in middle-childhood that are direct and that are indirect via reduced maternal wellbeing and parent–child relationship quality (acting in isolation and sequentially). In doing so we have identified that children residing in households in the lowest two quintiles for household income at age 3 have more than twice the odds of experiencing mental health difficulties at age 6 than children from all other quintiles combined. Our analysis identified that, in isolation, reduced maternal wellbeing and child parent relationship quality each explain approximately 11% and 18% of this difference respectively, and that a combined pathway with of these mediators acting sequentially accounts for close to a third (30.89%).

While the association between household income and children’s mental health is well recognised, the causal pathways which may account for this association have received relatively limited research attention to date and the majority of the evidence which does exist on the mediating effects of parent–child relationships currently comes from within the United States [5]. These findings therefore contribute to a small but growing number of studies which suggest that household income affects children’s mental health via pathways other than parenting style and maternal wellbeing. Nevertheless, they suggest that the intermediary effect of both maternal wellbeing and parent child relationships is sizeable and are the first to demonstrate this in a sample of children from the UK.

Policies which support household income have a key role to play in improving the mental wellbeing of children from disadvantaged backgrounds. Currently, close to 1 in 4 children in Scotland live in poverty, and the cost-of-living crisis has driven already disadvantaged households further into hardship [44]. The majority of these children are in a home where at least one parent is working [45]. Despite this, financial support for families experiencing poverty has been capped in recent years and has been evidenced to be increasingly insufficient [46]. An increase in childhood mental health inequalities has been observed alongside this [47]. Our results indicate that through failing to prevent families from experiencing economic hardship and limiting overall household income for those that do, these policies are not fulfilling their potential to support children’s mental health. They also suggest that any income increases which are gained through the welfare system may not be of optimal benefit to children’s mental wellbeing if the impact of welfare policies on both mother’s wellbeing and their relationships with their children are not considered. We’ve found in GUS children that those residing in households with higher incomes have better mental health outcomes, partly because of the benefits that higher incomes have for mother’s wellbeing and parenting style. However, it cannot be assumed that income increases through policies such as Universal Credit will have the same benefits if they come with conditions which negatively impact upon those pathways. Numerous reports have highlighted the detrimental mental health impacts the UK’s existing social security system holds for claimants, with stringent conditionality identified as one of the main drivers of this [48]. Conversely, studies of unconditional cash transfers for children have been associated with significant and long-lasting benefits for their mental health, particularly when these are introduced early [49]. However, a mediation analyses of one of these studies identified that income changes in isolation did not significantly mediate these improved mental health outcomes for children [21]. Rather it was increased parental supervision and an improvement in parent–child relationships due to reduced time constraints within the family which were central [21]. Recent years have seen growing interest in a variety of welfare models which aim to remove the harsh conditionality currently associated with Universal Credit, such as a Universal Basic Income, or a Guaranteed Minimum Income [49].Research exploring the potential benefits of models such as these and other forms unconditional income support (such as the recently introduced Scottish Child Payment [50]) for the mental health of recipient’s children would therefore be of significant value.

### Strengths and limitations

The implications of our findings must be considered in relation to the design of the study. The GUS study is a large nationally representative sample of the Scottish population, which is a key strength of our analysis, and we used weights and imputation to account for attrition and item missingness. However, this is unlikely to have completely addressed attrition among families facing the most severe socioeconomic hardship, who are known to have been over-represented among those lost to follow-up within GUS [27]. Future longitudinal studies should therefore look to methods, such as oversampling, which promote better representation of this sociodemographic group within research and avoid any systematic bias which may result from their exclusion. It must also be noted that accurately measuring household income through interview questions, as were employed in GUS, has previously shown to be challenging and the validity of this measure must therefore be considered carefully [51]. The dichotomisation of the mediators may have introduced measurement error, which could lead to an underestimation of the mediating pathways, although as noted in the methods, the cut-off for child-parent relationship quality has been shown to be meaningful through validation. Moreover, the use of only selected measures from the DASS within GUS also limits the inferences which can be drawn from our results, as use of these questions in isolation has not previously been validated. Nonetheless, well validated measures for each of our other variables of interest were utilised, namely the CPRS-SF [36] and the parent-report SDQ [52].

In addition, our study is clearly observational in nature and does not directly test an intervention. As a result, conclusions about causality are limited and further interventional studies are needed to strengthen the causal inferences which can be drawn from these observed associations. It must also be noted that we have examined just two mediating pathways for the purposes of this analysis. It is also possible that for some families causal pathways act in the reverse direction from those proposed by the family stress model. For example, while total SDQ scores have previously been shown to have good sensitivity in identifying children who are likely to have a mental health difficulty [32], they could also be picking up pre-existing neurodevelopmental conditions or other forms of behavioural difficulties [53]. There is evidence to show that for some families having a child with these additional needs prevents, delays or reduces a parent’s ability to return to work, thereby limiting their income [54, 55]. Having a child with additional support needs can also influence parental mental health [56], as well as parent–child relationships [57]. If such reverse causation is simultaneously occurring, with such difficulties contributing to low income, which in turn causes further mental health or behavioural difficulties, in a vicious cyclical nature, through looking at this relationship in only one direction we are likely to have underestimated the overall effects of low income.

Finally, while we were able to account for the majority of factors which have previously been shown to confound the relationship between household income and children’s mental well-being over time, there are likely to have been other confounding factors which we were unable to control for in the present study. Specifically, the statistical method used in this paper cannot account for exposure induced mediating confounding, which may create bias in our estimation of the indirect and direct effects. For example, the impact of relationship breakdown, poverty stigma [58] and of parent’s social capital [5] on children’s and parents’ mental wellbeing has previously been shown to be important, but it was not possible to account for these using the method employed.

## Conclusions

Overall, our results support the assertion that the significant rise in poverty across the UK and the dramatic increase in the prevalence of childhood mental health difficulties are unlikely to be unrelated. As such, any strategy aimed at tackling the UK’s childhood mental health crisis may not be successful if the social context in which it has emerged is ignored. These findings should be of interest to policymakers who have the ability to redistribute incomes through employment, taxation or welfare policies. In light of these, and other recent findings, the introduction of measures which alleviate economic hardship for families with young children in particular must be considered. Such policy changes should focus on how financial support can be delivered in a manner that does not adversely affect either parent’s wellbeing or parent–child relationships, while providing income at a level that minimises financial stress. Finally, upwards of two thirds of the effect of household income on children’s mental health was not explained by either parental mental health or perceived parent–child relationship quality. Further research attention to explore other pathways through which incomes effect children’s mental health (and other outcomes) might point towards other areas for intervention. Specifically, future research could explore additional mediators, such as community support, access to mental health services, or housing stability, to better understand the mechanisms linking income and child mental health. It should also explore the effects of exposure to low income at other ages, with extended follow-up periods (for example into adolescence or adulthood), in order to provide valuable insights surrounding longer-term effects. In addition, while we would primarily advocate for upstream interventions, research attention should also be paid toward the development of parenting interventions which are designed to support and bolster parent wellbeing and parent–child relationship quality in the context of such economic adversity. Given the long-term mental health, physical health and social risks associated with experiencing childhood mental health difficulties greater efforts to address child poverty are required.

## Supplementary Information

Below is the link to the electronic supplementary material.

Supplementary file1 (DOCX 182 KB)

## Data Availability

The Growing Up in Scotland data that support the findings of this study are not openly available due to reasons of sensitivity, but are available from the UK Data Service upon successful application (https://beta.ukdataservice.ac.uk/datacatalogue/).

## References

[1] Minnis H, Pollard A, Boyd K, Davidson J, Godfrey K, Green J, ... Viner R (2024) Prioritising early childhood to promote the nation’s health, wellbeing and prosperity

[2] Gariepy G, Elgar FJ, Sentenac M, Barrington-Leigh C (2017) Early-life family income and subjective well-being in adolescents. PLoS One 12(7):e0179380 28715418 10.1371/journal.pone.0179380 PMC5513414

[3] Reiss F (2013) Socioeconomic inequalities and mental health problems in children and adolescents: a systematic review. Soc Sci Med 90:24–31 23746605 10.1016/j.socscimed.2013.04.026

[4] Kinge JM, Øverland S, Flatø M, Dieleman J, Røgeberg O, Magnus MC, ... Torvik FA (2021) Parental income and mental disorders in children and adolescents: prospective register-based study. Int J Epidemiol 50(5):1615–1627 10.1093/ije/dyab066 PMC8580274 33975355

[5] Cooper K, Stewart K (2021) Does household income affect children’s outcomes? A systematic review of the evidence. Child Indic Res 14(3):981–1005

[6] Hillier-Brown F, Thomson K, Mcgowan V, Cairns J, Eikemo TA, Gil-Gonzále D, Bambra C (2019) The effects of social protection policies on health inequalities: evidence from systematic reviews. Scandinavian J Public Health 47(6):655–665 10.1177/1403494819848276 31068103

[7] Simpson J, Albani V, Bell Z, Bambra C, Brown H (2021) Effects of social security policy reforms on mental health and inequalities: a systematic review of observational studies in high-income countries. Soc Sci Med 272:113717 33545493 10.1016/j.socscimed.2021.113717

[8] Nordenmark M, Strandh M, Layte R (2006) The impact of unemployment benefit system on the mental well-being of the unemployed in Sweden, Ireland and Great Britain. Europ Societies 8(1):83–110

[9] Williams E (2021) Unemployment, sanctions and mental health: the relationship between benefit sanctions and antidepressant prescribing. J Soc Policy 50(1):1–20

[10] Pearce A, Dundas R, Whitehead M, Taylor-Robinson D (2019) Pathways to inequalities in child health. Arch Dis Child 104(10):998–1003 30798258 10.1136/archdischild-2018-314808 PMC6889761

[11] Sutherland H (2006) Can child poverty be abolished? Promises and policies in the UK. Econ Labour Relations Rev 17(1):7–31

[12] Kraemer HC, Stice E, Kazdin A, Offord D, Kupfer D (2001) How do risk factors work together? Mediators, moderators, and independent, overlapping, and proxy risk factors. Am J Psychiatry 158(6):848–856 11384888 10.1176/appi.ajp.158.6.848

[13] Masarik AS, Conger RD (2017) Stress and child development: A review of the family stress model. Curr Opin Psychol 13:85–90 28813301 10.1016/j.copsyc.2016.05.008

[14] Conger RD, Ge X, Elder GH, Lorenz FO, Simons RL (1994) Economic stress, coercive family process, and developmental problems of adolescents. Child Dev 65(2):541–561. 10.2307/1131401 8013239

[15] Conger RD, Conger KJ, Elder GH Jr, Lorenz FO, Simons RL, Whitbeck LB (1992) A family process model of economic hardship and adjustment of early adolescent boys. Child Dev 63(3):526–541 1600820 10.1111/j.1467-8624.1992.tb01644.x

[16] Lai ET, Schlüter DK, Lange T, Straatmann V, Andersen AMN, Strandberg-Larsen K, Taylor-Robinson D (2020) Understanding pathways to inequalities in child mental health: a counterfactual mediation analysis in two national birth cohorts in the UK and Denmark. BMJ Open 10(10):e040056 33046476 10.1136/bmjopen-2020-040056 PMC7552869

[17] Noonan K, Burns R, Violato M (2018) Family income, maternal psychological distress and child socio-emotional behaviour: Longitudinal findings from the UK Millennium Cohort Study. SSM-Population Health 4:280–290 29854912 10.1016/j.ssmph.2018.03.002 PMC5976845

[18] Ho LLK, Li WHC, Cheung AT, Luo Y, Xia W, Chung JOK (2022) Impact of poverty on parent–child relationships, parental stress, and parenting practices. Front Public Health 10:849408 10.3389/fpubh.2022.849408 PMC9081330 35548071

[19] Kwon B, Lee IH, Lee G (2023) Maternal predictors of children’s mental health in low-income families: A structural equation model. Int J Ment Health Nurs 32(1):162–171 36109880 10.1111/inm.13071

[20] Akee R, Copeland W, Costello EJ, Simeonova E (2018) How does household income affect child personality traits and behaviors? Am Econ Rev 108(3):775–827 29568124 10.1257/aer.20160133 PMC5860688

[21] Costello EJ, Compton SN, Keeler G, Angold A (2003) Relationships between poverty and psychopathology: A natural experiment. JAMA 290(15):2023–2029 14559956 10.1001/jama.290.15.2023

[22] Henry A, Wernham T (2024) Child poverty: trends and policy options. London: institute for fiscal studies. Available at: https://ifs.org.uk/publications/child-poverty-trends-and-policy-options. Accessed 20 Mar 2025

[23] Child Poverty Action Group (2024) The cost of child poverty in 2023. Available from: https://cpag.org.uk/news/cost-child-poverty-2023. Accessed Mar 2025

[24] Balasundaram P, Avulakunta ID. (2023) Human Growth and Development. In: StatPearls [Internet]. Treasure Island (FL): StatPearls Publishing 33620844

[25] Bradshaw P, Tipping S, Marryat L et al (2007) Growing up in Scotland sweep 1–2005 user guide. Edinburgh: Scottish Centre for Social Research. Available at: https://growingupinscotland.org.uk/data-documentation. Accessed Mar 2025

[26] VanderWeele TJ, Chiba Y (2014) Sensitivity analysis for direct and indirect effects in the presence of exposure-induced mediator-outcome confounders. Epidemiol. Biostat. Public Health 11(2). 10.2427/9027 10.2427/9027 PMC4287391 25580387

[27] Barnes M, Chanfreau J, Tomaszewski W (2010) Growing up In Scotland: The circumstances of persistently poor children. National Centre for Social Research: Edinburgh

[28] Goodman R, Ford T, Simmons H, Gatward R, Meltzer H (2018) Using the strengths and difficulties questionnaire (SDQ) to screen for child psychiatric disorders in a community sample. Br J Psychiatry 177(6):534–539 10.1192/bjp.177.6.534 11102329

[29] Deighton J, Croudace T, Fonagy P, Brown J, Patalay P, Wolpert M (2014) Measuring mental health and wellbeing outcomes for children and adolescents to inform practice and policy: a review of child self-report measures. Child Adolesc Psychiatry Ment Health 8:1–14 24834111 10.1186/1753-2000-8-14 PMC4022575

[30] Husky MM, Otten R, Boyd A, Pez O, Bitfoi A, Carta MG, ... Kovess-Masfety V (2018) Psychometric properties of the Strengths and Difficulties Questionnaire in children aged 5–12 years across seven European countries. Euro J Psychological Assess 36(1):65–76. 10.1027/1015-5759/a000489

[31] Goodman A, Goodman R (2009) Strengths and difficulties questionnaire as a dimensional measure of child mental health. J Am Acad Child Adolesc Psychiatry 48(4):400–403 19242383 10.1097/CHI.0b013e3181985068

[32] Goodman A, Goodman R (2011) Population mean scores predict child mental disorder rates: validating SDQ prevalence estimators in Britain. J Child Psychol Psychiatry 52(1):100–108 20722879 10.1111/j.1469-7610.2010.02278.x

[33] Crawford JR, Henry JD (2003) The depression anxiety stress scales (DASS): Normative data and latent structure in a large non-clinical sample. Br J Clin Psychol 42(2):111–131 12828802 10.1348/014466503321903544

[34] Lovibond SH, Lovibond PF (1995) Manual for the depression anxiety stress scales, 2nd edn. Psychology Foundation of Australia, Sydney

[35] Pianta RC (1992) Child-parent relationship scale. Unpublished measure, University of Virginia, p 427

[36] Driscoll K, Pianta RC (2011) Mothers’ and fathers’ perceptions of conflict and closeness in parent-child relationships during early childhood. J Early Childhood Infant Psychol 7:1–24

[37] Dyer WJ, Kaufman R, Fagan J (2017) Father–child closeness and conflict: Validating measures for nonresident fathers. J Fam Psychol 31(8):1074 29309190 10.1037/fam0000384

[38] Hernán MA, Hernández-Díaz S, Werler MM, Mitchell AA (2002) Causal knowledge as a prerequisite for confounding evaluation: an application to birth defects epidemiology. Am J Epidemiol 155(2):176–184 11790682 10.1093/aje/155.2.176

[39] IBM Corp. Released 2023. IBM SPSS Statistics for Windows, Version 29.0.2.0 Armonk, NY: IBM Corp

[40] Van Buuren S, Groothuis-Oudshoorn K (2011) mice: Multivariate imputation by chained equations in R. J Stat Softw 45:1–67

[41] Kowarik A, Templ M (2016) Imputation with the R Package VIM. J Stat Softw 74:1–16

[42] Steen J, Loeys T, Moerkerke B, Vansteelandt S (2017) Medflex: an R package for flexible mediation analysis using natural effect models. J Stat Softw 76:1–4636568334

[43] VanderWeele T (2015) Explanation in causal inference: methods for mediation and interaction. Oxford University Press

[44] Jospeh Rowntree Foundation (2024) UK Poverty 2024. Online: Joseph Rowntree Foundation. Retrieved from: https://www.jrf.org.uk/uk-poverty-2024-the-essential-guide-to-understanding-poverty-in-the-uk

[45] Scottish Government National Statistics (2021) Poverty and Income Inequality in Scotland 2017–2020. Retrieved online: https://data.gov.scot/poverty/2021/

[46] Lammasniemi L (2019) The benefit cap and infliction of poverty. J Soc Welf Fam Law 41(3):368–371

[47] Fairchild G (2019) Mind the gap: evidence that child mental health inequalities are increasing in the UK. Eur Child Adolesc Psychiatry 28(11):1415–1416 31605207 10.1007/s00787-019-01418-1

[48] Dwyer P, Scullion L, Jones K, McNeill J, Stewart AB (2020) Work, welfare, and wellbeing: The impacts of welfare conditionality on people with mental health impairments in the UK. Soc Policy Administration 54(2):311–326

[49] Wilson N, McDaid S (2021) The mental health effects of a Universal Basic Income: A synthesis of the evidence from previous pilots. Soc Sci Med 287:114374 34534779 10.1016/j.socscimed.2021.114374

[50] Congreve E, Connolly K, Harrison J, Kumar A, McGregor PG, Mitchell M (2024) The impact of using an income supplement to meet child poverty targets: evidence from Scotland. J Soc Policy 53(4):933–949

[51] Oakes JM, Andrade KE (2017) The measurement of socioeconomic status. Methods Soc Epidemiol 18:23–42

[52] Silva TB, Osório FL, Loureiro SR (2015) SDQ: discriminative validity and diagnostic potential. Front Psychol 6:811 26113840 10.3389/fpsyg.2015.00811 PMC4462033

[53] Goodman A, Lamping DL, Ploubidis GB (2010) When to use broader internalising and externalising subscales instead of the hypothesised five subscales on the Strengths and Difficulties Questionnaire (SDQ): data from British parents, teachers and children. J Abnorm Child Psychol 38:1179–1191 20623175 10.1007/s10802-010-9434-x

[54] Cidav Z, Marcus SC, Mandell DS (2012) Implications of childhood autism for parental employment and earnings. Pediatrics 129(4):617–623 22430453 10.1542/peds.2011-2700 PMC3356150

[55] Amaro J, Costa R, Popovic M, Maule MM, Mehlum IS, Lucas R (2024) Association of child neurodevelopmental or behavioural problems with maternal unemployment in a population-based birth cohort. Soc Psychiatry Psychiatr Epidemiol 59(4):643–655 36967439 10.1007/s00127-023-02464-6 PMC10960748

[56] Lach LM, Kohen DE, Garner RE, Brehaut JC, Miller AR, Klassen AF, Rosenbaum PL (2009) The health and psychosocial functioning of caregivers of children with neurodevelopmental disorders. Disabil Rehabil 31(9):741–752 19736648 10.1080/08916930802354948

[57] Potter-Dickey A, Letourneau N, de Koning APJ (2020) Associations between neurodevelopmental disorders and attachment patterns in preschool-aged children: Systematic review. Curr Dev Disord Rep 7:277–289

[58] Inglis G, Jenkins P, McHardy F, Sosu E, Wilson C (2023) Poverty stigma, mental health, and well-being: A rapid review and synthesis of quantitative and qualitative research. J Commun Appl Soc Psychol 33(4):783–806

