# Supplementary material for: Household income and children’s mental health outcomes: the mediating role of maternal wellbeing and parent–child relationship quality
Source: Eur Child Adolesc Psychiatry. 2025 Jun 7;34(11):3601–11. doi: 10.1007/s00787-025-02765-y (PMC12647339; doi:10.1007/s00787-025-02765-y)
Supplement: Supplementary file 1 — Supplementary file1 (DOCX 182 KB) [file 787_2025_2765_MOESM1_ESM.docx]

Supplementary Figure 1. The Family Stress Model and Proxy Measures*


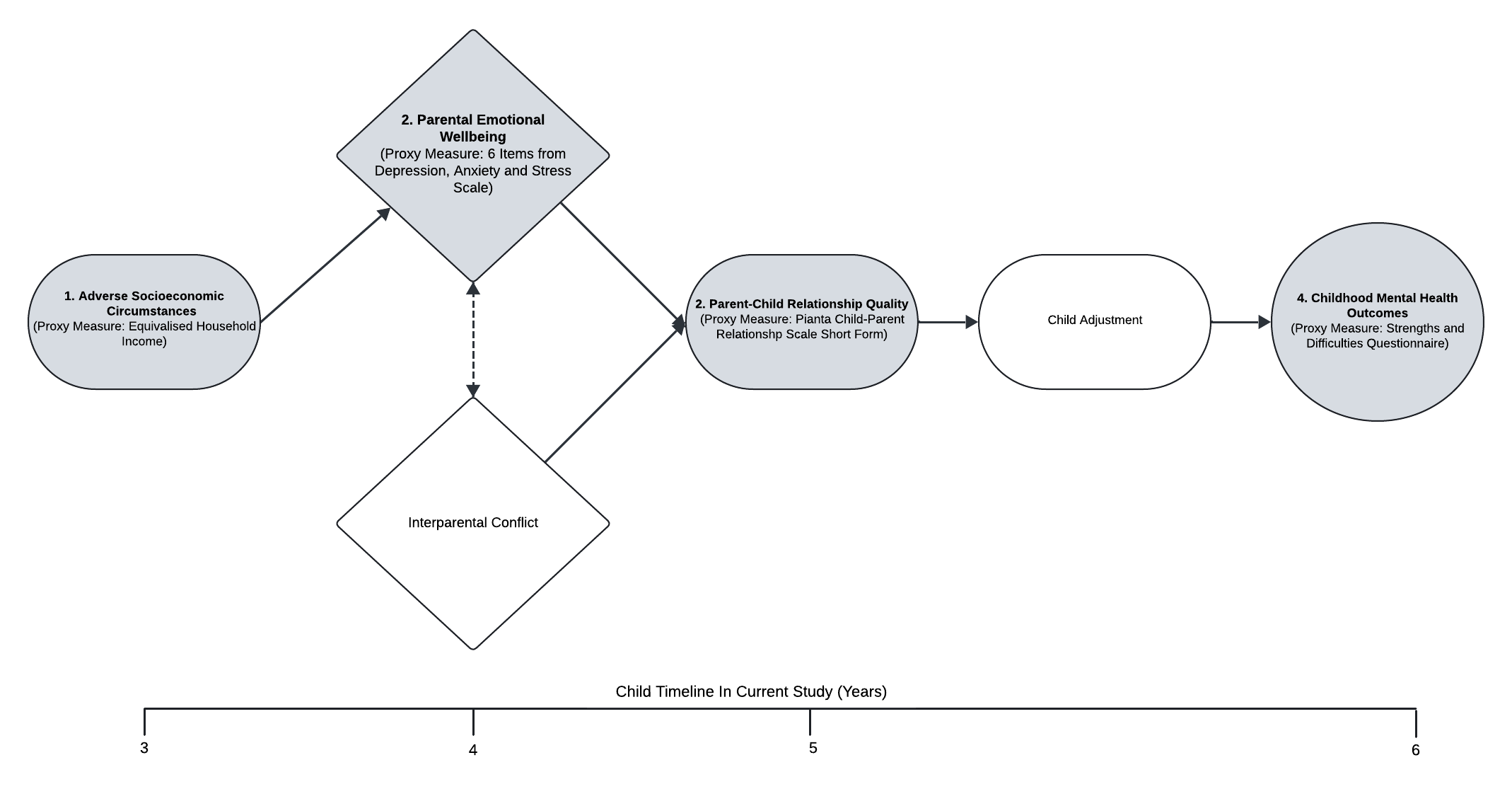


(*Components of the Family Stress Model included in current study highlighted in bold)

| Supplementary Table 1. Growing Up in Scotland Response Rates | | | |
| --- | --- | --- | --- |
| **Sweep** | **Sample achieved** | **Response rate** | |
|  |  | As % of issued | As % of sweep 1 |
| 1 | 5217 | 80% | 100% |
| 2 | 4512 | 88% | 86% |
| 3 | 4193 | 90% | 80% |
| 4 | 3994 | 91% | 77% |
| 5 | 3883 | 92% | 74% |
| 6 | 3657 | 87% | 70% |

|  |  |  |  |  |  |
| --- | --- | --- | --- | --- | --- |
